# Supplementary material for: Stacking Model‐Based Classifiers for Dealing With Multiple Sets of Noisy Labels
Source: Biom J. 2025 Mar 12;67(2):e70042. doi: 10.1002/bimj.70042 (PMC11898607; doi:10.1002/bimj.70042)
Supplement: Supplementary file 1 — Supporting Information [file BIMJ-67-e70042-s001.zip › density-based-ensemble-model-main/competitors_real_data/netlab-master/netlab-master/help/mlppak.htm]

Netlab Reference Manual mlppak


# mlppak

## Purpose

Combines weights and biases into one weights vector.

## Synopsis

```
w = mlppak(net)
```

## Description

`w = mlppak(net)` takes a network data structure `net` and
combines the component weight matrices bias vectors into a single row
vector `w`. The facility to switch between these two
representations for the network parameters is useful, for example, in
training a network by error function minimization, since a single
vector of parameters can be handled by general-purpose optimization
routines.

The ordering of the paramters in `w` is defined by

```
  w = [net.w1(:)', net.b1, net.w2(:)', net.b2];
```

where `w1` is the first-layer weight matrix, `b1` is the
first-layer bias vector, `w2` is the second-layer weight matrix,
and `b2` is the second-layer bias vector.

## See Also

`mlp`, `mlpunpak`, `mlpfwd`, `mlperr`, `mlpbkp`, `mlpgrad`

---

**Pages:**
Index


---

Copyright (c) Ian T Nabney (1996-9)
